# Podocan and Adverse Clinical Outcome in Patients Admitted With Suspected Acute Coronary Syndromes

**DOI:** 10.3389/fcvm.2022.867944

**Published:** 2022-05-20

**Authors:** Thomas Andersen, Thor Ueland, Pål Aukrust, Dennis W. Nilsen, Heidi Grundt, Harry Staines, Frederic Kontny

**Affiliations:** ^1^Department of Anesthesiology, Stavanger University Hospital, Stavanger, Norway; ^2^Department of Clinical Science (K2), University of Bergen, Bergen, Norway; ^3^Research Institute of Internal Medicine, Oslo University Hospital Rikshospitalet, Oslo, Norway; ^4^Institute of Clinical Medicine, University of Oslo, Oslo, Norway; ^5^K.G. Jebsen Thrombosis Research and Expertise Center, University of Tromsø, Tromsø, Norway; ^6^Section of Clinical Immunology and Infectious Diseases, Oslo University Hospital Rikshospitalet, Oslo, Norway; ^7^Department of Cardiology, Stavanger University Hospital, Stavanger, Norway; ^8^Department of Clinical Science, University of Bergen, Bergen, Norway; ^9^Department of Pulmonology, Stavanger University Hospital, Stavanger, Norway; ^10^Sigma Statistical Services, Balmullo, United Kingdom; ^11^Drammen Heart Center, Drammen, Norway

**Keywords:** extracellular matrix, chest pain, acute coronary syndrome, risk factors, biomarkers, Podocan

## Abstract

**Background:**

Markers of bone and extracellular matrix (ECM) remodeling may be associated with adverse outcomes in atherosclerotic cardiovascular disease. Podocan is a newly discovered ECM glycoprotein, previously not studied in a chest pain population. We wanted to study the association between Podocan levels on admission and the risk of adverse outcomes in a chest pain population with suspected acute coronary syndromes.

**Methods:**

A total of 815 patients from the Risk markers in Acute Coronary Syndrome (RACS) trial with suspected coronary chest pain were followed for 7 years. Blood samples were taken immediately after inclusion and stored in the biobank. Associations between Podocan and endpoints were assessed with Cox proportional hazards analyses.

**Results:**

The median admission level of Podocan was 0.674 ng/ml (0.566–0.908 ng/ml). No significant association was found between Podocan quartile levels and all-cause death, neither at 1 year nor 2- or 7-years follow-up (*p* > 0.05 for all). Furthermore, no significant association could be shown between Podocan and cardiac death, myocardial infarction (MI), stroke, or the composites of all-cause death/MI/stroke or cardiac death/MI/stroke (*p* > 0.05 for all). Similarly, in a subgroup of patients with Troponin T-positive (*n* = 432) there was no significant association between Podocan and any of the outcome measures (*p* > 0.05 for all endpoints and points in time).

**Conclusion:**

Podocan, a novel ECM biomarker, is not associated with all-cause mortality or other major cardiovascular adverse events in patients admitted with acute chest pain suspected to be of coronary origin.

**Clinical Trials.gov Identifier:**

NCT00521976.

## Introduction

Biomarkers have emerged as a key tool in the current management of patients with the acute coronary syndrome (ACS), both concerning diagnosis (e.g., troponins) and risk assessment (e.g., Estimated Glomerular Filtration Rate [eGFR] and Brain Natriuretic Peptide [BNP]) (1). Our understanding of the pathophysiological basis for ACS is also evolving as new biomarkers emerge and show new processes to be central both in disease development and progression. Sometimes this may eventually lead to new therapeutic strategies as the case has been in the unravelment of the central role of inflammation in ACS (2). Markers of bone metabolism and extracellular matrix (ECM) proteins may be associated with adverse outcomes in both ischemic heart disease and heart failure, but their role and underlying mechanisms are yet to be fully understood (3–6).

Podocan, a recently discovered ECM glycoprotein in the small leucin-rich repeat proteoglycan (SLRP) family, binds collagen type 1, the most abundant collagen type in atherosclerotic plaques (7, 8). It was originally discovered in the kidney basal membrane, but has also been found in myocardial tissue, aortic tissue, and coronary atheroma as well as in vascular smooth muscle cells (SMC) (7, 9, 10). As the biomarker field progresses, several aspects of the cascade of atherosclerosis are illuminated. Similar to inflammation, fibrosis is an integral part of atherosclerosis involving ECM remodeling and its bidirectional interaction with inflammation as an important mediator (4). Podocan has been shown to inhibit excessive arterial repair after injury in preclinical trials (11). This could suggest that Podocan may have a protective role against scar formation and fibrosis after myocardial injury. Contrary to this, abstracts have shown an increased risk of adverse events from higher Podocan levels in patients with angiographic evidence of coronary artery disease (12, 13) potentially reflecting that excessive Podocan levels may impair tissue healing As the role of ECM and fibrosis is still explored, Podocan as a novel and unestablished biomarker in this field should be investigated further in larger clinical cohorts. At present, there are only abstracts published on the association between Podocan and clinical outcomes in coronary artery disease (CAD) and no studies investigating Podocan in an undifferentiated chest pain population.

The objective of the current study was to evaluate the role of Podocan as a new biomarker of prognosis in an acute chest pain population suspected of having ACS.

## Materials and Methods

### Design and Study Population

The Risk in Acute Coronary Syndrome (RACS–ClinicalTrials.gov Identifier: NCT00521976) was a single-center, prospective, cohort study on consecutive patients admitted with a history of chest pain, or symptoms suggestive of ACS to Stavanger University Hospital, Stavanger, Norway, in the period from November 2002 to September 2003 (14). Exclusion criteria were age under 18 years, unwillingness or incapacity to provide informed consent and prior inclusion in the same study. The planned follow-up was 24 months, but the study was later expanded to include 7 years of follow-up. Baseline clinical data were collected during hospitalization and follow-up was done by patient interviews by phone at 30 days, 6 and 12 months, and 2 and 7 years. In cases where data collection was not possible to obtain by phone, this was retrieved through contact with the patient's general practitioner, nursing home, or close relatives. Hospital records were used to confirm data. Written informed consent was obtained from all patients, and the study was approved by the Regional Board of Research Ethics and the Norwegian Health authorities and conducted in accordance with the Helsinki Declaration. Blood samples were stored in a biobank approved by the Norwegian Health Authorities.

### Endpoints

The following endpoints were recorded in the study: All-cause death, cardiac death, myocardial infarction (MI), stroke, the composite of all-cause death, MI and stroke as well as the composite of cardiac death, MI, and stroke. Cardiac death was defined as death after definite MI, or chest pain over 20 min, or a history of ischemic heart disease and no other clear cause of death (15). MI was defined as symptoms of coronary ischemia with rising and fall of Troponin T (TnT) with levels ≥ 0.05 ng/ml. For the composite endpoints, only the first MI or stroke was recorded. The term ACS in this study includes ST-elevation MI (STEMI), Non–ST-elevation MI (NSTEMI), and unstable angina pectoris (UAP). STEMI was defined as ST-elevation in ECG and TnT>0.05 ng/ml (upper limit of normal at the study laboratory during this study period). NSTEMI was defined as chest pain without ST elevation in ECG and a TnT level > 0.05 ng/ml. UAP was defined as chest pain without ST-elevation and with TnT levels < 0.05 ng/ml. Non–ACS was defined as all other conditions, such as arrhythmias and unspecified chest pain, without ECG changes or positive TnT. A TnT positive subgroup was used for stratified analysis, rather than the patients with adjudicated ACS. This was chosen because modern assays are more sensitive and some of the patients with intermediary TnT levels on the contemporary assay would probably be regarded as MI today. To increase sensitivity in our analysis, we used a cut-off value of TnT > 0.01 ng/ml (the lower level of detection of the analysis used) to identify our subgroup.

### Laboratory Analysis

Baseline laboratory variables were collected from hospital records. Data on BNP, high sensitivity C-Reactive Protein (hsCRP), and TnT analyses are reported earlier together with information concerning blood sampling, transport, and storage (16).

Podocan was analyzed in blood sampled immediately upon hospital admission with a commercially available enzyme-linked immunosorbent assay-based analysis of ethylene diamine-tetra-acetic acid (EDTA)-plasma (Abbexa Ltd, Cambridge, United Kingdom, Cat# abx370876, RRID AB_2904494) at our cooperating research laboratory at Rikshospitalet, Oslo, Norway, in 2021. Intra- and inter-assay coefficients of variations for Podocan were <10%.

### Statistical Analysis

Baseline characteristics are presented by Podocan quartiles. Categorical variables are shown as frequency and percentage and tested across quartiles of Podocan using Chi-Square tests. Continuous variables are presented as a median and interquartile range and compared across quartiles of Podocan by Kruskal-Wallis tests. Biomarkers, such as BNP, were analyzed as quartiles. Survival analysis is presented as Kaplan-Meier plots grouped by quartiles of Podocan and equality of survival curves tested by log-rank tests. Receiver Operating Characteristics (ROC) curves and Area Under ROC (AUROC) were used to assess Podocan's ability to predict endpoints. Univariable Cox regression assessed Podocan quartiles' independent relation to endpoints before the primary analysis, a multivariable Cox proportional hazards regression model, with stepwise adjustment of variables. In the first step, relevant confounding variables were assessed, and only significant variables were included in the next step, introducing Podocan. The potential confounders were; age, sex, diabetes mellitus, hypertension, current smoking, dyslipidemia, prior heart disease (angina or MI), heart failure, prior medication use (statins, beta blockers, angiotensin converting enzyme inhibitors (ACEI) or angiotensin receptor blockers (ARB), diuretics and acetylsalicylic acid (ASA), discharge diagnosis (STEMI/NSTEMI/UAP/Non–ACS), primary revascularization within 50 days of index event, as well as quartiles of BNP, eGFR (Modification of Diet in Renal Disease [MDRD]) and hsCRP, as well as TnT>0.01 ng/ml. A two-sided *p* < 0.05 was considered statistically significant. As this study is exploratory in origin, no adjustments were introduced for multiple comparisons. With no published clinical studies on Podocan in chest pain patients, estimating event rates for quartiles of Podocan would be impossible beforehand, and hence no sample size calculation was done. All available patients from the RACS-material were analyzed in our study. All analyses were performed using SPSS version 25 (IBM, Armonk, New York, USA).

## Results

### Admission Levels of Podocan and Baseline Characteristics

A total of 871 patients were enrolled in the RACS study and samples for Podocan measurement were available for 815 patients (93.6%). There was no loss to follow-up. The median level of Podocan in the study was 0.674 ng/ml (0.566–0.908). The distribution of Podocan is shown in Supplementary Figure S1. The baseline characteristics of the population are given in Table 1. There was no significant difference in any of the baseline characteristics between quartiles of Podocan. During 7 years of follow-up, there were 315 deaths, 187 MIs, and 50 strokes for patients with known Podocan values. The frequencies of all outcomes are shown in Table 2. Frequencies of 7-years outcomes by discharge diagnosis are provided in Supplementary Table S1.

**Table 1:** Baseline characteristics of the study population.

	**Characteristics**	**Podocan (ng/mL)**					
		**Quartile1**	**Quartile2**	**Quartile3**	**Quartile4**	***p*–value**	**Total**
		***n* = 203**	***n* = 201**	***n* = 206**	***n* = 205**		***n* = 815**
		**0.30–0.56**	**0.57–0.67**	**0.67–0.91**	**0.91–23.06**		**0.30–23.06**
Demographics						
	Age, years, median (q1-q3)	71.8 (60.1–80.5)	72.6 (59.6–82.8)	72.3 (59.8–79.0)	73.4 (57.3–82.2)	0.657^†^	72.6 (59.0–81.2)
	Male, n (%)	130 (64.0)	124 (61.7)	129 (62.6)	117 (57.1)	0.503^*^	500 (61.4)
Comorbidities						
	Diabetes mellitus type I or II, *n* (%)	26 (12.8)	33 (16.4)	23 (11.2)	31 (15.1)	0.421^*^	113 (13.9)
	Hypertension, *n* (%)	87 (42.9)	89 (44.3)	91 (44.2)	76 (37.1)	0.403^*^	343 (42.1)
	Current smoking, *n* (%)	56 (27.6)	46 (22.9)	58 (28.2)	49 (23.9)	0.530^*^	209 (25.6)
	Dyslipidemia, *n* (%)	102 (50.3)	98 (48.8)	106 (51.5)	98 (47.8)	0.887^*^	404 (49.6)
	Prior MI or angina, *n* (%)	108 (53.2)	124 (61.7)	111 (53.9)	115 (56.1)	0.301^*^	458 (56.2)
	Prior heart failure, *n* (%)	50 (24.6)	61 (30.4)	57 (27.7)	55 (26.8)	0.636^*^	223 (27.4)
Medication prior to admission						
	Statins, *n* (%)	71 (35.0)	70 (34.8)	75 (36.4)	67 (32.7)	0.887^*^	283 (34.7)
	Betablocker, *n* (%)	71 (35.0)	80 (39.8)	76 (36.9)	67 (32.7)	0.494^*^	294 (36.1)
	ACEI/ARB, *n* (%)	63 (31.0)	73 (36.3)	72 (35.0)	69 (33.7)	0.712^*^	277 (34.0)
	Diuretics, *n* (%)	61 (30.1)	67 (33.3)	59 (28.6)	67 (32.7)	0.708^*^	254 (31.2)
	ASA, *n* (%)	77 (37.9)	79 (39.3)	78 (37.9)	77 (37.6)	0.985^*^	311 (38.2)
Index diagnosis						0.489^*^	
	UAP, *n* (%)	16 (7.9)	22 (11.0)	19 (9.2)	17 (8.3)		74 (9.1)
	NSTEMI, *n* (%)	56 (27.6)	48 (23.9)	66 (32.0)	67 (32.7)		237 (29.1)
	STEMI, *n* (%)	32 (15.8)	25 (12.4)	33 (16.0)	28 (13.7)		118 (14.5)
	NON–ACS, *n* (%)	99 (48.8)	106 (52.7)	88 (42.7)	93 (45.4)		386 (47.4)
Treatment						
	Primary revascularization within 50 days, *n* (%)	52 (25.6)	35 (17.5)	52 (25.2)	44 (21.5)	0.171^*^	183 (22.5)
Biomarkers						
	eGFR, ml/min/1.73 m^2^, median (q1-q3)	63.5 (49.3–74.9)	64.2 (47.1–75.2)	64.1 (50.8–74.8)	61.8 (49.4–76.9)	0.988^†^	63.3 (49.2–75.3)
	hs-CRP, mg/L, median (q1-q3)	3.2 (1.7–9.8)	4.1 (1.8–13.5)	4.3 (1.7–10.1)	4.1 (1.7–18.0)	0.278^†^	4.0 (1.7–13.3)
	BNP, pg/mL, median (q1-q3)	101.0 (33.0–330.0)	90.0 (38.0–237.0)	110.0 (35.5–387.5)	91.0 (30.5–330.5)	0.735^†^	97.0 (34.0 −316.0)
	Maximum TnT release > 0.01 ng/mL, n (%)	107 (52.7)	94 (46.8)	119 (57.8)	112 (54.6)	0.155^*^	432 (53.0)

* *Chi-squared test*.

† *Kruskal-Wallis test*.

*MI, Myocardial Infarction; ACEI, Angiotensin Converting Enzyme Inhibitor; ARB, Angiotensin Receptor Blocker; ASA, Acetylsalicylic acid; UAP, Unstable Angina Pectoris; NSTEMI, Non–ST-elevation Myocardial Infarction; STEMI, ST-elevation Myocardial Infarction; Non–ACS, Non– Acute Coronary Syndrome; eGFR, estimated Glomerular Filtration Rate; hs-CRP, High sensitivity C-Reactive Protein; BNP, Brain Natriuretic Peptide; TnT, Troponin T*.

**Table 2:** Frequencies of outcomes.

**Outcome**	**Year**	**Total population, *n* (%)**	**TnT+ population, *n* (%)**
All-cause death	1	94 (11.5)	80 (18.5)
	2	127 (15.6)	96 (22.2)
	7	315 (38.7)	204 (47.2)
Cardiac death	1	63 (7.7)	57 (13.2)
	2	81 (9.9)	68 (15.7)
MI	1	82 (10.1)	70 (16.2)
	2	141 (17.3)	113 (26.2)
	7	187 (22.9)	137 (31.7)
Stroke	1	15 (1.8)	8 (1.9)
	2	25 (3.1)	13 (3.0)
	7	50 (6.1)	25 (5.8)
Death/MI/Stroke	1	157 (19.3)	127 (29.4)
	2	237 (29.1)	176 (40.7)
	7	393 (48.2)	251 (58.1)
Cardiac death/MI/Stroke	1	134 (16.4)	110 (25.5)
	2	204 (25.0)	156 (36.1)

### Podocan and Its Relation to Adverse Clinical Outcomes

**Figure 1:**
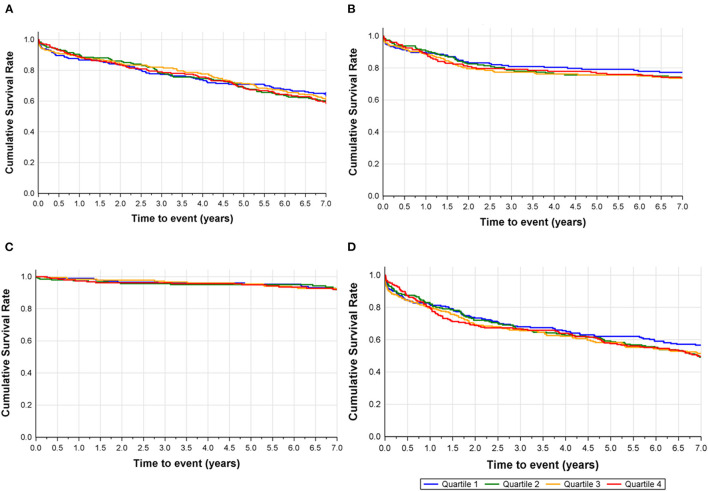
Kaplan-meier plots of endpoints by quartile of podocan, **(A)** all-cause death, **(B)** myocardial infarction, **(C)** stroke and **(D)** composite of all-cause death, myocardial infarction, or stroke.

The incidence of all-cause death did not differ between quartiles of Podocan at 7 years (*p* = 0.706). Kaplan-Meier plots for the event rates by quartiles of Podocan are shown in Figure 1. The log-rank test did not show any significant differences in the survival curves for the Podocan quartiles (*p* = 0.839), and the results of ROC analysis indicated no potential role for Podocan to predict fatal outcomes (AUROC=0.512, *p* = 0.574). Neither univariable nor multivariable Cox regression analyses could demonstrate any significant association between Podocan and all-cause mortality (*p* = 0.840 and 0.992, respectively) with similar results at 1- and 2-years follow-up.

Concerning the composite of all-cause death/MI/stroke or the individual endpoints of MI or stroke alone, no difference was seen in univariable or multivariable analyses between Podocan quartiles at 7 years or other time points (*p* > 0.05 for all). The same results were found for cardiac death and the composite of cardiac death/MI/stroke at 1 and 2 years. Details are given in Table 3.

**Table 3:** Association between podocan and all endpoints in the total population.

**Time**	**Endpoint**	**Univariable cox**		Multivariable cox^*^	
		**HR (Q4 vs. Q1)**	***p*-value**	**HR (Q4 vs. Q1)**	***p*-value**
1 year	All–cause death	0.89 (0.51–1.55)	0.682	0.76 (0.43–1.35)	0.349
	Cardiac death	0.76 (0.39–1.50)	0.432	0.71 (0.35–1.43)	0.340
	MI	1.05 (0.58–1.93)	0.864	0.97 (0.52–1.81)	0.934
	Stroke	2.41 (0.47–12.44)	0.292	2.16 (0.42–11.12)	0.358
	Death/MI/stroke	1.09 (0.70–1.70)	0.690	1.07 (0.68–1.68)	0.774
	Cardiac death/MI/stroke	1.12 (0.69–1.83)	0.638	1.07 (0.65–1.76)	0.790
2 years	All–cause death	1.06 (0.66–1.72)	0.802	0.89 (0.54–1.48)	0.662
	Cardiac death	0.97 (0.54–1.76)	0.925	0.73 (0.39–1.35)	0.312
	MI	1.15 (0.71–1.84)	0.573	1.09 (0.67–1.76)	0.741
	Stroke	1.15 (0.39–3.41)	0.805	1.11 (0.37–3.31)	0.851
	Death/MI/stroke	1.18 (0.82–1.70)	0.367	1.20 (0.83–1.74)	0.338
	Cardiac death/MI/stroke	1.23 (0.82–1.83)	0.314	1.20 (0.80–1.80)	0.385
7 years	All–cause death	1.14 (0.83–1.56)	0.417	1.00 (0.72–1.39)	0.988
	MI	1.15 (0.76–1.73)	0.523	1.00 (0.66–1.53)	0.992
	Stroke	1.09 (0.50–2.39)	0.830	1.08 (0.49–2.36)	0.853
	Death/MI/stroke	1.19 (0.90–1.58)	0.231	1.12 (0.84–1.51)	0.439

* *Model adjusted for significant confounding variables among age, sex, diabetes mellitus, hypertension, current smoking, dyslipidemia, prior heart disease, heart failure, prior medication, index diagnosis, primary revascularization within 50 days, BNP, eGFR, CRP, and peak TnT*.

*MI, Myocardial Infarction*.

### Podocan in the TnT Positive Subpopulation

Of the 815 patients included in the study, 432 (53.0%) had TnT >0.01 ng/ml. The median level of Podocan was 0.686 ng/ml (0.567–0.924 ng/ml). During 7 years of follow-up, 204 all-cause deaths, 137 MIs, and 25 strokes occurred in this subgroup. As in the total population, baseline characteristics did not differ across quartiles of Podocan (*p* > 0.05 for all, details in Supplementary Table S2). Moreover, similar to the total population, no association could be demonstrated for Podocan with all-cause death or any other outcome measures at any time point, neither in univariable nor in multivariable Cox regression (*p* > 0.05 for all, details in Table 4). Due to zero events in a combination of variables, the hazard ratio for a 2-years stroke in the multivariable Cox regression could not be computed.

**Table 4:** Association between Podocan and all endpoints in the TnT positive subpopulation.

**Time**	**Endpoint**	**Univariable cox**		Multivariable cox^*^	
		**HR (Q4 vs.Q1)**	***p*-value**	**HR (Q4 vs.Q1)**	***p*-value**
1 year	All-cause death	0.70 (0.38–1.32)	0.270	0.62 (0.32–1.18)	0.144
	Cardiac death	0.73 (0.35–1.50)	0.392	0.65 (0.31–1.37)	0.255
	MI	0.98 (0.51–1.86)	0.942	1.01 (0.53–1.95)	0.972
	Stroke	1.89 (0.17–20.83)	0.604	1.74 (0.16–19.22)	0.651
	Death/MI/stroke	0.87 (0.53–1.44)	0.589	0.89 (0.54–1.48)	0.650
	Cardiac death/MI/stroke	0.97 (0.57–1.66)	0.904	0.98 (0.57–1.69)	0.941
2 years	All-cause death	0.74 (0.42–1.31)	0.298	0.61 (0.33–1.11)	0.105
	Cardiac death	0.71 (0.37–1.40)	0.325	0.63 (0.31–1.28)	0.202
	MI	0.95 (0.56–1.62)	0.846	1.05 (0.61–1.82)	0.867
	Stroke	1.43 (0.24–8.54)	0.697	NA^†^	NA^†^
	Death/MI/stroke	0.91 (0.59–1.40)	0.663	0.94 (0.60–1.48)	0.801
	Cardiac death/MI/stroke	0.98 (0.62–1.57)	0.944	1.12 (0.69–1.80)	0.648
7 years	All-cause death	0.91 (0.61–1.35)	0.633	0.80 (0.53–1.21)	0.285
	MI	0.98 (0.60–1.59)	0.927	0.94 (0.57–1.55)	0.794
	Stroke	1.16 (0.35–3.81)	0.805	1.15 (0.35–3.78)	0.816
	Death/MI/stroke	0.94 (0.66–1.35)	0.744	0.89 (0.61–1.30)	0.547

* *Model adjusted for significant confounding variables among age, sex, diabetes mellitus, hypertension, current smoking, dyslipidemia, prior heart disease, heart failure, prior medication, index diagnosis, primary revascularization within 50 days, BNP, eGFR, CRP, and peak TnT*.

† *Coefficients did not converge, calculation not possible*.

*MI, Myocardial Infarction; NA, Not applicable*.

## Discussion

In this first study in the literature to assess Podocan in patients with suspected ACS, we did not find that Podocan was associated with all-cause or cardiac mortality, or with MI, stroke, or composites of these individual endpoints. Similar results were found in patients with elevated troponins.

To the best of our knowledge, no peer-reviewed articles on the role of Podocan in cardiovascular (CV) disease have so far been published. However, there are some abstracts on this topic available in the literature (12, 13, 17–19). Data from these indicate an association of Podocan with major adverse CV events among patients with angiographically documented CAD, which is in contrast to our results in the present study. However, the inherent shortcomings of a scientific report in abstract format make the reconciliation of the overall findings in these series difficult. As there are no studies investigating levels of Podocan in the general population or chest pain/ACS, we cannot compare the levels from our material to others, neither in the total material or subgroup.

Podocan is part of the SLRP-family, consisting of five “branches” based on conservation, protein, and genetic similarities (20). Podocan was discovered as the first member of a new class (Class 5) of non–canonical SLRPs, differing in the C-terminal cysteine clusters, but sharing 20 leucine-rich repeat sections with strong homology to the canonical class 1 and 2 (20). Podocan also shares functional properties with other SLRPs by binding collagen type 1 and inhibiting cell growth through increased expression of p21 (7). Preclinical research suggests that Podocan acts through inhibition of the wingless T-Cell Factor (Wnt-TCF)-pathway (11). The Wnt-TCF pathway is a regulatory pathway for smooth muscle cell growth and subsequently, Podocan is part of the regulatory process for fibrosis in blood vessels. The same study showed that mice deficient in Podocan had increased, prolonged, and excessive neointima formation after injury (11). Similarly, human smooth muscle cells with overexpression of Podocan had reduced activity of the WnT-TCF pathway, paving the way for a theory of Podocan being protective of fibrosis and possibly a marker of beneficial outcomes after atherosclerotic events. This was supported by an abstract showing increased cardiac hypertrophy in Podocan deficient mice after induced pressure overload (18). Contrary to this, abstracts investigating patients with angiographic confirmed CAD showed a positive association between measurable Podocan and increased risk of adverse CV events, hence questioning the protective role of Podocan (12, 13). Another study failed to find a significant association between Podocan and pulse-wave velocity, a known predictor of CV risk (21). In conclusion there is a scarcity of, and conflicting evidence of the effects and association of Podocan to risk in CV disease. While experimental data may suggest a protective role of Podocan in CV disease, the natural dynamics and pathophysiological effects are still uncertain.

There may be several reasons for Podocan not showing an association to outcomes in our study. As atherosclerosis and the evolution of CV disease after an index event is vastly complex, there is always the possibility of the preclinical finding being tempered by other processes or feedback loops in clinical practice. There is also the possibility of the murine experiments not being generalizable to humans, and Podocan not having the effects in humans as found in mice (22). As stated, the dynamics of Podocan are unknown, and hence our finding of a lack of association may be a question of sampling time after the index event. Also, as in any quantitative study, our lack of significant findings may be a question of sample size, and our study being underpowered to find the existing associations. Additionally, it is important to underscore that whereas inhibition of fibrogenesis and ECM remodeling may be beneficial by protecting against excessive fibrosis, too much inhibition by, e.g., high levels of Podocan may attenuate infarct healing following MI and may, therefore, be harmful, illustrating the dual roles of a mediator such as Podocan. Finally, the balance between canonical and non–canonical Wnt signaling is finely tuned by other secreted Wnt agonists and antagonists and local effects in the vasculature may not necessarily be reflected by systemic levels. Thus, our findings do not mean Podocan has no role in ACS progression but rather that it does not exhibit prognostic characteristics.

### Strengths and Limitations

Including over 800 patients, this is a relatively large biomarker study. The study population was old with a median age of over 70 years. However, with a consecutive enrolment, this study population is representative of patients admitted into emergency rooms in our area. As our study was originally designed to follow patients for 2 years, and only later expanded to 7 years, we only have follow-up data for cardiac death for up to 2 years. There was no loss to follow-up, but no cause specific death was adjudicated for the 7-years data. The blood samples have been stored in a biobank for up to 18 years before the present analyses. Effects of long-term storage on Podocan are not known, and no studies investigating this are published to date. As all samples in our study were equally exposed, the effects on results should be random and negligible. We believe that our biobank material is still able to give insights into any associations between Podocan and endpoints, that would in any instance need to be verified in other studies. Although the ethnicity in the study area is mainly Scandinavian, the lack of data on ethnicity may have hampered our conclusion.

Medication and treatment are major determinants of outcomes after ACS. As an exploratory study, we did not aim to adjust for all confounders and quantify the effect of Podocan on outcomes. As such, only medication before admission and invasive treatment within 50 days were included in the statistical model. There was no change in standards of treatment during the inclusion period.

There is a lack of research on the dynamics of Podocan in peripheral blood in the process of vascular injury and healing, hence the optimal time window for sampling is unknown. Even though our results could be affected by this, the complete lack of signal in our material makes Podocan unlikely to be a good predictor of risk.

## Conclusion

In this first study on patients with acute chest pain admitted to the hospital for suspected ACS, Podocan, a novel glycoprotein and Wnt pathway inhibitor were not associated with all-cause mortality or CV morbidity. More research is needed to confirm our findings.

## Data Availability Statement

The raw data supporting the conclusions of this article will be made available by the authors, without undue reservation.

## Ethics Statement

The studies involving human participants were reviewed and approved by Regional Etisk Komite Vest University of Bergen, Medical Faculty, PB 7804, 5020 Bergen. The patients/participants provided their written informed consent to participate in this study.

## Author Contributions

TA, FK, DN, TU, and PA contributed to conception and design of the study. HG organized the database. TU performed laboratory analyses. HS performed the statistical analysis. TA wrote the first draft of the manuscript. All authors contributed to manuscript revision, read, and approved the submitted version.

## Funding

This study was funded through a personal research grant to TA from Helse Vest, Grant Number 912294. The laboratory analyses for this study are in part funded through project funding from Stavanger Universitetssjukehus, Grant Number 501814.

## Conflict of Interest

DN has received support for travel and/or attending meetings from Stavanger Health Research in the last 36 months. HS is employed by Sigma Statistical Services. The remaining authors declare that the research was conducted in the absence of any commercial or financial relationships that could be construed as a potential conflict of interest.

## Publisher's Note

All claims expressed in this article are solely those of the authors and do not necessarily represent those of their affiliated organizations, or those of the publisher, the editors and the reviewers. Any product that may be evaluated in this article, or claim that may be made by its manufacturer, is not guaranteed or endorsed by the publisher.
