# Correction: Podocan and adverse clinical outcome in patients admitted with suspected acute coronary syndromes

**DOI:** 10.3389/fcvm.2025.1650098

**Published:** 2025-07-07

**Authors:** Thomas Andersen, Thor Ueland, Pål Aukrust, Dennis W. Nilsen, Heidi Grundt, Harry Staines, Frederic Kontny

**Affiliations:** ^1^ Department of Anesthesiology, Stavanger University Hospital, Stavanger, Norway; ^2^ Department of Clinical Science (K2), University of Bergen, Bergen, Norway; ^3^ Research Institute of Internal Medicine, Oslo University Hospital Rikshospitalet, Oslo, Norway; ^4^ Institute of Clinical Medicine, University of Oslo, Oslo, Norway; ^5^ K.G. Jebsen Thrombosis Research and Expertise Center, University of Tromsø, Tromsø, Norway; ^6^ Section of Clinical Immunology and Infectious Diseases, Oslo University Hospital Rikshospitalet, Oslo, Norway; ^7^ Department of Cardiology, Stavanger University Hospital, Stavanger, Norway; ^8^ Department of Clinical Science, University of Bergen, Bergen, Norway; ^9^ Department of Pulmonology, Stavanger University Hospital, Stavanger, Norway; ^10^ Sigma Statistical Services, Balmullo, United Kingdom; ^11^ Drammen Heart Center, Drammen, Norway

**Keywords:** extracellular matrix, chest pain, acute coronary syndrome, risk factors, biomarkers, Podocan

**A Correction on**
Podocan and adverse clinical outcome in patients admitted with suspected acute coronary syndromes By Andersen T, Ueland T, Aukrust P, Nilsen DW, Grundt H, Staines H and Kontny F (2022). Front Cardiovasc Med. 9:867944. doi: 10.3389/fcvm.2022.867944

Affiliation “Department of Clinical Science (K2), University of Bergen, Bergen, Norway” was omitted from the paper. This affiliation has now been added for author Thomas Andersen.

The original version of this article has been updated.

