# Supplementary material for: Podocan and Adverse Clinical Outcome in Patients Admitted With Suspected Acute Coronary Syndromes
Source: Front Cardiovasc Med. 2022 May 20;9:867944. doi: 10.3389/fcvm.2022.867944 (PMC9163367; doi:10.3389/fcvm.2022.867944)
Supplement: Supplementary file 1 [file Table_1.PDF]

Supplemental table S1: 7-year outcome by discharge diagnosis

**Outcome**	**Discharge diagnosis**			
	**Non-ACS**	**STEMI**	**NSTEMI**	**UAP**
All cause death, n (%)	113 (29,3)	33 (28,0)	112 (47,3)	57 (77,0)
MI, n (%)	53 (13,7)	21 (17,8)	87 (36,7)	26 (35,1)
Stroke, n (%)	25 (6,5)	6 (5,1)	12 (5,1)	7 (9,5)

Abbreviations: ACS - Acute Coronary Syndrome, STEMI - ST elevation myocardial infarction, NSTEMI - Non-ST elevation myocardial infarction, UAP - Unstable angina pectoris
