# Supplementary material for: Podocan and Adverse Clinical Outcome in Patients Admitted With Suspected Acute Coronary Syndromes
Source: Front Cardiovasc Med. 2022 May 20;9:867944. doi: 10.3389/fcvm.2022.867944 (PMC9163367; doi:10.3389/fcvm.2022.867944)
Supplement: Supplementary file 2 [file Table_2.PDF]

Table S2: Baseline characteristics for the TnT positive subpopulation

	Characteristics	Podocan (ng/mL) Quartile1 n = 108 0.30 - 0.57	Quartile2 n = 109 0.57 - 0.69	Quartile3 n = 108 0.69 - 0.92	Quartile4 n = 107 0.94 - 6.86	p-value	Total n = 432 0.30 - 6.86
Demographics							
	Age, years, median (q1-q3)	73.6 (63.5 - 82.1)	74.8 (68.2 - 83.2)	74.1 (62.4 - 81.2)	75.6 (57.7 - 82.7)	0.669†	74.6 (63.0 - 82.6)
	Male, n (%)	69 (63.9)	74 (67.9)	74 (68.5)	68 (63.6)	0.806*	285 (66.0)
Comorbidities							
	Diabetes mellitus type I or II, n (%)	21 (19.4)	22 (20.2)	16 (14.8)	15 (14.0)	0.520*	74 (17.1)
	Hypertension, n (%)	49 (45.4)	46 (42.2)	51 (47.2)	36 (33.6)	0.189*	182 (42.1)
	Current smoking, n (%)	30 (27.8)	27 (24.8)	37 (34.3)	29 (27.1)	0.452*	123 (28.5)
	Dyslipidemia, n (%)	44 (40.7)	53 (48.6)	50 (46.3)	47 (43.9)	0.684*	194 (44.9)
	Prior MI or angina, n (%)	59 (54.6)	70 (64.2)	58 (53.7)	57 (53.3)	0.309*	244 (56.5)
	Prior heart failure, n (%)	36 (33.3)	42 (38.5)	33 (30.6)	27 (25.2)	0.205*	138 (31.9)
Medication prior to admission	Statins, n (%)	31 (28.7)	36 (33.0)	37 (34.3)	28 (26.2)	0.540*	132 (30.6)
	Betablocker, n (%)	35 (32.4)	37 (33.9)	33 (30.6)	32 (29.9)	0.918*	137 (31.7)
	ACEI/ARB, n (%)	41 (38.0)	35 (32.1)	41 (38.0)	36 (33.6)	0.734*	153 (35.4)
	Diuretics, n (%)	42 (38.9)	38 (34.9)	34 (31.5)	35 (32.7)	0.679*	149 (34.5)
	ASA, n (%)	37 (34.3)	40 (36.7)	39 (36.1)	32 (29.9)	0.717*	148 (34.3)
Index diagnosis						0.540*	
	UAP, n (%)	16 (14.8)	24 (22.0)	18 (16.7)	16 (15.0)		74 (17.1)
	NSTEMI, n (%)	56 (51.9)	56 (51.4)	59 (54.6)	65 (60.8)		236 (54.6)
	STEMI, n (%)	33 (30.6)	28 (25.7)	30 (27.8)	26 (24.3)		117 (27.1)
	NON ACS, n (%)	3 (2.8)	1 (0.9)	1 (0.9)	0 (0.00)		5 (1.2)
Treatment							
	Primary revascularization within 50 days, n (%)	47 (43.5)	38 (34.9)	42 (38.9)	39 (36.5)	0.582*	166 (38.4)
Biomarkers							
	eGFR, ml/min/1.73m^2, median (q1-q3)	61.0 (46.9 - 73.9)	62.3 (41.4 - 77.0)	62.0 (47.9 - 74.7)	59.5 (45.7 - 77.8)	0.981†	61.4 (45.0 - 75.4)
	hs-CRP, mg/L, median (q1-q3)	3.8 (1.8 - 13.9)	6.0 (2.4 - 13.8)	6.0 (2.3 - 12.7)	7.0 (2.6 - 25.0)	0.061†	5.7 (2.2 - 17.1)
	BNP, pg/mL, median (q1-q3)	215.0 (49.0 - 511.0)	164.0 (60.0 - 487.0)	147.0 (42.0 - 549.0)	158.0 (46.0 - 384.0)	0.765†	175.0 (49.0 - 480.0)

* Chi-squared test. † Kruskal-Wallis test. Abbreviations: MI - Myocardial Infarction; ACEI - Angiotensin Converting Enzyme Inhibitor; ARB - Angiotensin Receptor Blocker; ASA - Acetylsalicylic acid; UAP - Unstable Angina Pectoris; NSTEMI - Non ST-elevation Myocardial Infarction; STEMI - ST-elevation Myocardial Infarction; Non ACS - Non Acute Coronary Syndrome; eGFR - estimated Glomerular Filtration Rate; hs-CRP - High sensitivity C-Reactive Protein; BNP - Brain Natriuretic Peptide
