# Supplementary figures and images for: Podocan and Adverse Clinical Outcome in Patients Admitted With Suspected Acute Coronary Syndromes
Source: Front Cardiovasc Med. 2022 May 20;9:867944. doi: 10.3389/fcvm.2022.867944 (PMC9163367; doi:10.3389/fcvm.2022.867944)

**Supplemental Figure S1: Distribution of log-transformed Podocan**

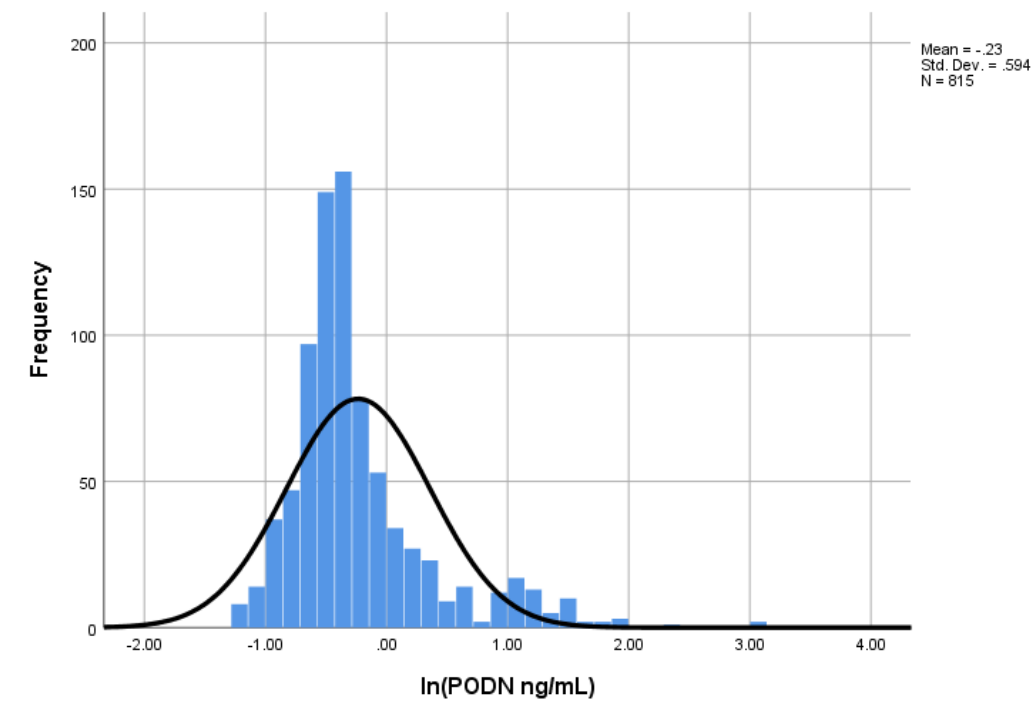

Supplement: Supplementary file 3 [file Image_1.PDF]
